# Postoperative use of fitness trackers for continuous monitoring of vital signs: a survey of hospitalized patients

**DOI:** 10.1007/s10877-025-01273-3

**Published:** 2025-03-06

**Authors:** Philipp Helmer, Sebastian Hottenrott, Kathrin Wienböker, Jürgen Brugger, Christian Stoppe, Benedikt Schmid, Peter Kranke, Patrick Meybohm, Michael Sammeth

**Affiliations:** 1 Department of Anaesthesiology, Intensive Care, Emergency and Pain Medicine, University Hospital Würzburg, Oberdürrbacher Str. 6, 97080 Würzburg, Germany; 2 Department of Applied Sciences and Health, Coburg University of Applied Sciences and Art, Friedrich-Streib-Str. 2, 96450 Coburg, Germany

**Keywords:** Anesthesia, Health tracker, Smartwatch, Ward monitoring, Wearables, Compliance, Safety

## Abstract

Wearables and Internet of Things (IoT) technologies are increasingly incorporated into healthcare, including perioperative settings. These devices offer continuous non-invasive monitoring of vital signs, patient position, and mobilization. Nonetheless, there is currently little information about tolerance and acceptance of wearables in postoperative patients. We therefore assessed opinions and user experience in postoperative patients who used three popular fitness trackers during their entire hospital stay. Specifically, we evaluate the Apple Watch 7, Garmin Fenix 6 Pro, and Withings ScanWatch. We used an investigator-designed patient questionnaire with 11 questions to quantify patient experience and opinions regarding inpatient and home monitoring. Secondarily, we evaluated compliance and the incidence of associated adverse events during daily patient visits. Data were analyzed using descriptive statistics and non-parametric tests. The majority of the answers to the questions (82.1%) were rated positively defined as Likert-Scale Scores 4 or 5 by the 33 analyzed patients, ranging between 72.7 and 97.0% agreement rate. Specific questions related to data sharing for research and overall user experience received high agreement rates (97.0 and 84.8%, respectively). Women reported slightly higher satisfaction with device comfort, as compared to men (LS-Score 4.8 vs. 4.0). No significant differences were found based on the device model or length of hospitalization. The use of wearable devices in healthcare is rated positively by postoperative inpatients, paving the way for future implementation of these devices in healthcare. However, besides validating the measurement accuracy and demonstrating clinical benefits, several regulatory hurdles must be overcome before implementing wearables in routine clinical care.

**Supplementary Information:**

The online version contains supplementary material available at 10.1007/s10877-025-01273-3.

## Introduction

Over the recent years, efforts towards integrating wearables and complementary Internet of Things (IoT) technologies in healthcare have substantially intensified, particularly in the perioperative setting [1]. These devices enable new possibilities, shifting the focus towards the continuous and non-invasive monitoring of vital signs [2] and allowing for further objective measurements [3, 4]. Traditional methods based on intermittent vital sign measuring can lead to critical gaps in patient monitoring, potentially delaying the detection of complications and therefore raising the rate of so-called “failure to rescue” events [5]. The continuous monitoring by wearables, however, possibly offers the potential to further close these gaps, providing real-time data that may encourage earlier interventions [1, 6].

Therefore, healthcare professionals strongly support the development of improved monitoring technologies: in a post-interventional survey that employed a continuous monitoring system based on wearables in a general ward, 97% of the surveyed healthcare professionals indicated that they foresee a future utility for systems along these lines [7]. A global survey of anesthesiologists sketches a similar picture, according to which the majority (91%) of the participants are convinced that continuous vital sign monitoring should be available on surgical wards, with 86% of them favoring wireless sensors over traditional systems [8]. Furthermore, 90% of the surveyed anesthesiologists stated that the deterioration of health conditions can be anticipated earlier by IoT technologies as compared to the current clinical standards. By all these means, particularly wearables—such as wrist-worn devices, smart patches and smart textiles—currently are at the forefront of the IoT revolution. These devices are advantageous not only because they are comfortable to wear, but also because they do not interfere with the user's everyday activities [4]. In a recent survey, the majority (71%) of anesthesiologists reported a preference for wrist-worn devices as a tool for patient monitoring [8]. Apart from professional opinions, regulatory aspects, existing technical limitations and questions about the reliability of the measurements, patient compliance and comfort are critical hurdles that need to be overcome before wearable devices can be routinely integrated as a clinical standard [9]. Despite the promising potential of tracker technologies, there is still only limited data available on inpatient satisfaction with wrist-worn devices, as highlighted by a recent systematic scoping review [10].

Therefore, we conducted a survey in hospitalized postoperative patients after using one out of three popular fitness trackers—Apple Watch 7, Garmin Fenix 6 Pro, and the Withings ScanWatch—during their entire hospital stay. We aim to evaluate patient compliance and satisfaction with these wrist-worn wearables based on their own user-experiences along with the incidence of associated adverse events.

## Methods

### Study design

The study protocol is in line with ethical, and scientific principles as defined in the Declaration of Helsinki (2013, Fortaleza), as well as with the guidelines of good clinical practice. Before trial enrollment, the approval of the study protocol was obtained from the ethics committee of the University of Würzburg in Germany (Ref. no. 145/21_c). Present study was conducted independently, without financial support or contributions by industrial partners, to prevent any potential conflicts of interest. This manuscript represents one of the secondary endpoints of the “Monitor project” (NCT05418881) and therefore by design comprises the same patient cohort investigated in our complementary manuscript [11].

The study enrollment started in May 2022 at the Department of Anaesthesiology, Intensive Care, Emergency, and Pain Medicine at the University Hospital Würzburg, Germany.

### Study procedure

In order to participate in the study, patients had to be capable of reading/understanding the German language and of comprehending the full orientation as well as the informed consent. Patients with allergies to any known components of the devices were excluded. Furthermore, critically ill patients defined as ASA V were excluded, as well as patients who had already participated in the study, patients with existing skin lesions on the forearms or patients considered ineligible for inclusion by the study physician. The study population targeted patients undergoing elective moderate to major surgery followed by hospitalization, as a sufficient device wear time was deemed necessary for a reliable questionnaire-based assessment. If a planned surgery eventually was not performed or discontinued, the corresponding patient was excluded secondarily since a prolonged hospital stay could no longer be expected.

Written informed consent has been obtained from all included patients prior to elective surgical procedures. Postoperatively, at the post anesthesia care unit, the patients were randomly assigned to receive one of three popular fitness trackers, with manufacturer dependent sizes and respectively weights: (i) Apple Watch 7 (45 × 38 mm, 38.8 g), (ii) Garmin Fenix 6 Pro (47 × 47 mm, 83 g), and (iii) Withings ScanWatch (38 × 38 mm, 58 g). Thereby the employed devices continuously measured vital signs including heart rate, respiratory rate and oxygen saturation in addition to daily steps.

When attaching the fitness tracker to the patients, the length of the wristbands was individually adapted for each test subject to ensure a comfortable placement of the device. Afterwards, all patients were instructed by the study team about the correct use of the devices. The participants also were encouraged to wear the devices permanently until hospital discharge and not to take them off by themselves (i.e., "auto-removal"), except under one of the following circumstances: showering, medical interventions such as surgical procedures, diagnostic imaging, or any interventions that require a high level of hygiene.

The study team carried out daily visits to each patient to check their status and furthermore to charge the batteries of the fitness tracker when needed and to record the compliance with wearing the device and associated adverse events. On the day of hospital discharge, the devices were collected and the questionnaire was completed together with the patients. In case of an early termination requested by the patient, the completion of the questionnaire was correspondingly preponed.

### Data acquisition

The primary objective of the present manuscript was to survey the opinion and satisfaction of postoperative patients using fitness trackers during their entire hospital stay. The secondary objectives were to evaluate the patient compliance and the incidence of device associated adverse events. The present manuscript is a secondary analysis of the monitor cohort, consequently, there is no calculation of the sample size specifically for this study. The evaluation tool employed in this study was a self-designed, non-validated questionnaire elaborated in the German language (translated questions in Table 1; original German version in Supplement). The responses by participants to the questionnaire statements were modeled by a Likert-Scale (LS) [12], which provided possible scores ranging from 1 ("strongly disagree"), over 2 ("disagree"), 3 ("neither agree nor disagree"), 4 (“agree”), to 5 (“strongly agree”), and an additional option of "not applicable" for denying the response. Employing this scale, the phrasing of all questions has been designed to ensure that a score of 5 consistently reflects a positive response whereas a score of 1 indicates a negative response. The questionnaires filled out by hand were collected and the patient responses transferred to Excel files, along with the respective patient's age and gender. Compliance was monitored by daily visits, documenting any deviations from the study protocol. To evaluate the safety of the use of wrist-worn devices, all device associated adverse events were recorded and classified according to the 'Common Terminology Criteria for Adverse Events' (CTCAE) vocabulary [13].

**Table 1:** The translated questionnaire

Q1	The health tracker did not hamper me during the day
Q2	The health tracker did not hamper me at night
Q3	The overall user experience was fine
Q4	I would also use comparable devices at home
Q5	I have no concerns regarding data privacy
Q6	I am not concerned about continuous monitoring of my health metrics
Q7	I would like my family doctor to be able to access the measurements just in my presence
Q8	I would like my family doctor to be able to access the measurements always and in real-time
Q9	I am willing to share health tracker data with researchers
Q10	I wish to use a health tracker during any future hospitalization
Q11	Automated alert systems for health emergencies at home would enhance my feeling of safety

All questions (Q1 to Q11) have been translated from their original version in German

### Statistical analyses

All statistical analyses have been performed employing the R platform (version 4.4.0). Consequently, statistical indicators of LS-Score distributions (i.e., the mean, the standard deviation and relative proportions) were calculated by the usual R functions (Table 2). Also the heatmap (Fig. 2), the box plot (Fig. 3a) and the stacked bar plot diagram (Fig. 4) were produced by the corresponding default R functions. In contrast, the spider plot diagram (Fig. 3b) was visualized by the *radarchart*() function provided by the FSMB package.

The average *mean*(*LS*) and the standard deviation *SD*(*LS*) of LS-Scores separately for each question in the survey were computed (see Sect. 3.1). In order to reflect the support for any of the items in the questionnaire, we computed across all participants the degree of dis-/agreement (rows "DisAgr" and "Agr" in Table 2) as the fraction of negative (LS ≤ 2) and respectively positive (LS ≥ 4) answers, also labeled as "acceptance” or “agreement”. A prior sub-analysis was planned for length of hospital stay, model of the respective devices and demographic attributes. The correlation between the duration of hospitalization and LS-Scores was evaluated by the *cor.test*() function, under the alternative hypothesis that the true correlation is unequal 0 (Sect. 3.2). Corresponding linear regression coefficients are reported as computed by the linear model *lm*(). Additionally, a two-tailed Wilcoxon rank-sum test was employed to assess the statistical significance of differences in the LS-Score distributions across different patient groups (i.e., patient sub-cohorts split by demographic attributes or the employed tracker device, Sect. 3.2) and questions or categories (Sect. 3.3). All p-values ≤ 0.05 were considered significant.

## Results

### Overview of the cohort

**Fig. 1:**
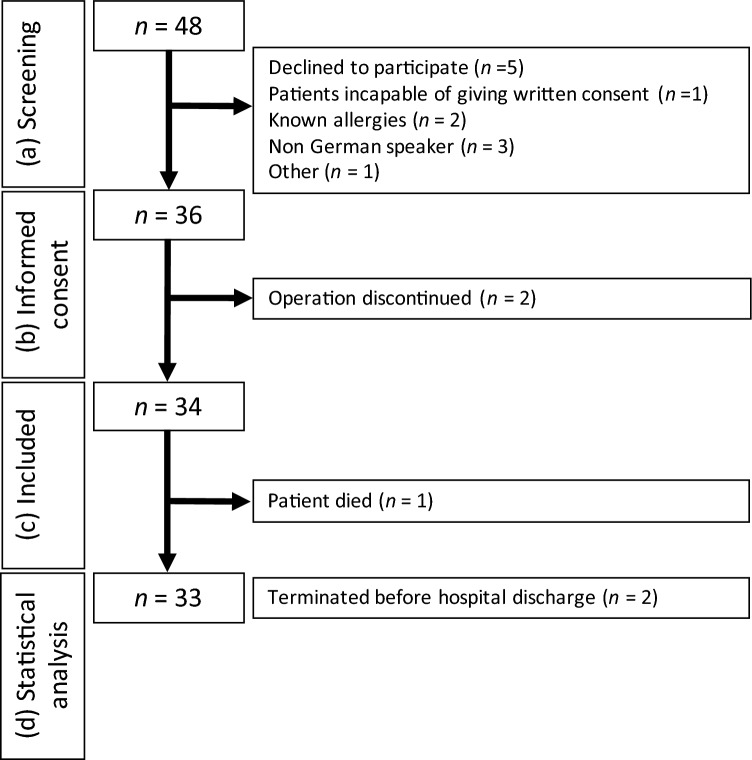
Study Flow chart. The flowchart provides the respective patient cohort sizes (*n*) separately for the different stages of **a** screening, **b** informed consent, **c** inclusion and **d** statistical analysis. At each stage, the numbers (*n*) of excluded patients are given with the corresponding reasons for exclusion

During the initial screening of 48 patients, five (10.4%) patients declined to participate in the trial and another seven (14.6%) patients were assigned at least one exclusion criteria. Of the 36 patients who provided written informed consent, surgery was discontinued in two patients and therefore no postoperative data could be collected, and one patient died (Fig. 1). The remaining 33 patients wore the fitness trackers throughout their entire hospital stay, however, two patients (6.1%) requested an early termination of their participation: one patient noted skin irritation, classified as CTCAE grade 1, and the other one felt discomfort from wearing the device. No auto-removal of a tracker by a patient except under the previously defined circumstances was recorded (Sect. 2.2).

The final study cohort included more female participants (54.5%) than male participants (45.5%), with a mean age of 65.4 years (ranging from 35 to 83 years). Considering all patients, the median time span of using the assigned fitness tracker was 7.6 days (min. 2 days, max. 21 days). After patient exclusions, slightly more patients using the Apple watch (*n* = 12) as compared to the Garmin (*n* = 11) or Withings (*n* = 10) were considered in the statistical analysis. From the 33 patients, 11 (33.3%) responded to all questions with LS-Score 5 ("strongly agree").

**Fig. 2:**
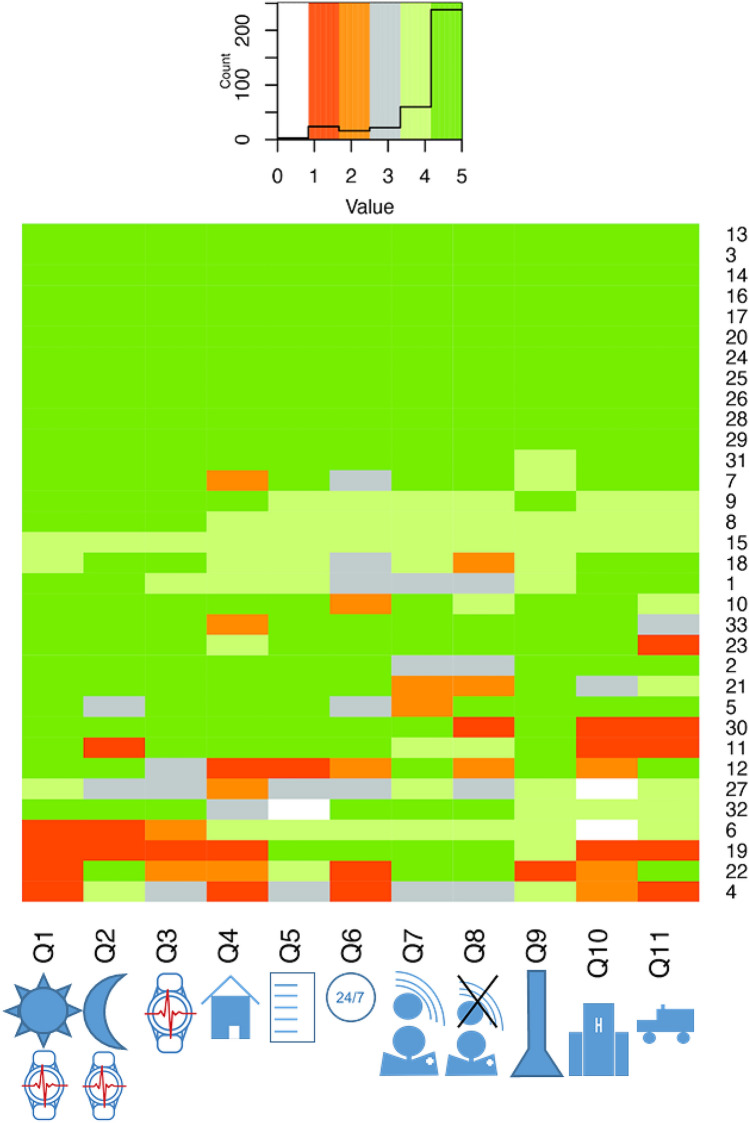
Heatmap of Likert-Scale stratified by patients and questions. Each patient's answer has been assigned a color according to the LS-Score (5 = green, 4 = light green, 3 = gray, 2 = orange, 1 = red, "not applicable" = white). The color-coded answers are shown at the respective coordinates spanned by the number of the respective question (Q1–Q11, x-axis) and by the participant number (1–33, y-axis). The histogram (top panel) summarizes the frequency of answers recorded for each LS-Score

Figure 2 presents a heatmap of all patient LS-Scores to the different questions. Overall, 298 out of the 363 patient answers (82.1%) were rated positively (LS-Score 4 and 5), 40 answers (11.0%) were rated negatively (LS-Score 1 and 2) and 22 responses (6.1%) were classified neither positive nor negative (LS-Score 3). The remaining three answers (0.8%) have been marked "not applicable" (Sect. 2.3).

Our results demonstrate that the willingness to share wearable data for research (Q9) yielded with 97.0% the highest degree of agreement. Focusing on the questions investigating possible anxieties regarding continuous monitoring (Q6) and the request of using wearables in any further hospitalization (Q10), they received a degree of agreement of merely 72.7% and therefore only a moderate support amongst the participants. Of note, by design with the possibilities of “not applicable” and “neither agree nor disagree”, values obtained for the degree of disagreement are not necessarily anti-correlated with the values calculated for the degree of agreement. To this end, our results show that the degree of disagreement was particularly high in the question about home monitoring (Q4), where 21.2% of the patients disagreed. Table 2 summarizes the results of the LS-Score and furthermore the rate of the degree of dis-/agreement. All questions regarding wearing comfort (Q1–Q3) and sharing data with the family doctor only in the presence of the patient (Q7) yield very similar results of LS-Score 4.42 to 4.45. In accordance with the agreement rate, the questions about data privacy (Q5) and sharing data with researchers (Q9) achieved the highest LS-Scores with the lowest SD: 4.53(0.88) and 4.55(0.79).

**Table 2:** Evaluation by question overview of *mean*(*LS*), subsuming the average LS-Score, and *SD*(*LS*) indicators, reporting the corresponding standard deviation, for each question (Q1 to Q11) in the survey. Answers ranked "not applicable" were excluded in the corresponding calculations. Furthermore, for each question the degree of agreement (row "Agr"), as by the proportion of LS ≥ 4, and the degree of disagreement (row "DisAgr" determined by the fraction of LS ≤ 2) is reported

	Q1	Q2	Q3	Q4	Q5	Q6	Q7	Q8	Q9	Q10	Q11
Mean (LS)	4.42	4.45	4.45	4.03	4.53	4.15	4.42	4.18	4.55	4.13	4.09
SD (LS)	1.32	1.23	1.09	1.40	0.88	1.25	0.90	1.16	0.79	1.41	1.42
Agr (%)	87.9	84.8	81.8	75.8	87.9	72.7	84.8	75.8	97.0	72.7	81.8
DisAgr (%)	12.1	9.1	9.1	21.2	3.1	12.1	6.1	12.1	3.0	19.4	15.2

### Subgroup analysis

**Fig. 3:**
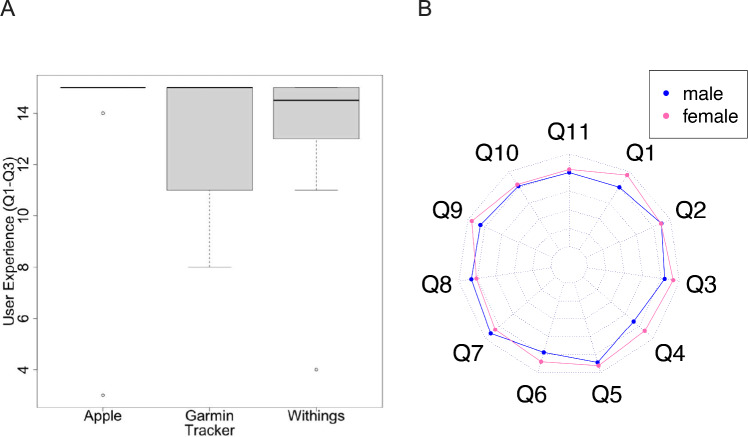
Analyses of systematic biases. **a** Boxplot of the distributions of overall user experience (summarized LS-Scores Q1 to Q3 with median marked as black line) (y-axis), segregated by the manufacturers of the three distinct fitness tracker models (x-categories). **b** Spider chart of gender-specific responses for Q1 to Q11. Females are depicted in pink, males in blue. Inner circle: "not applicable," followed by LS-Score

In order to investigate the presence of systematic biases in the participant answers, we first analyzed possible correlations between the assigned devices or the total time of the hospital stay on the reported wearing comfort and user experience, defined as summarized responses from Q1 to Q3. Figure 3a summarizes that LS-Scores did not exhibit significant deviations between fitness tracker models of different manufactures (*p* = *0.32; p* = *0.13; p* = *0.88*). There was no evidence for correlation between the duration of hospitalization, i.e. usage time, and the user experience (*r* =  *− 0.076; p* = *0.68*).

Next, we split our patient cohort according to different demographic attributes that were recorded for each patient. Subsequently, the distribution of LS values in both sub-cohorts was statistically compared with each other. Splitting at the average age of the cohort, we found for none of the questions in the survey a significant difference in the LS-Scores provided by younger (≤ 64 y.o., n = 18) and older (≥ 65 y.o., n = 15) participants (*p* = *[0.1;0.98]*).

In the absence of age-related biases in the responses, we next evaluated sex-specific differences. As depicted by Fig. 3b, women reported overall a significantly higher satisfaction with the wearing comfort of the tracker devices during the day (Q1) than men (*p* = *0.02;* mean LS-Score 4.8 *vs*. 4.0). Moreover, women also demonstrated a tendency to be more willing to use wearables at home (Q4) as compared to men (*p* = *0.16;* mean LS-Score 4.4 *vs*. 3.6), while they expressed not significantly less concern about data privacy (Q5: *p* > *0.1*; mean LS-Score 4.1 *vs*. 4.6).

### Categorical analysis

**Fig. 4:**
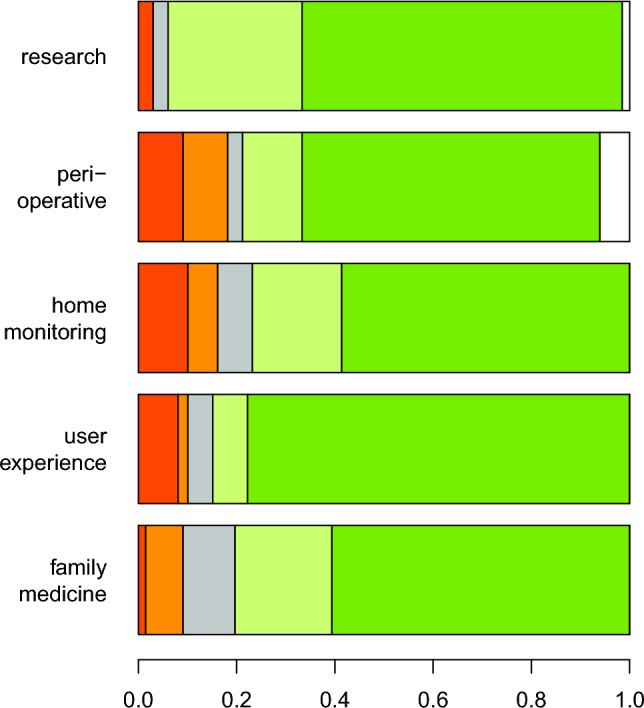
Categorical Segregation of the Questionnaire. Stacked bar plots visualize the proportion of LS-Scores (x-axis) in each of the considered five categories (y-categories): "research" = {Q5,Q9}, “perioperative use” = {Q10}, “home monitoring” = {Q4,Q6,Q11}, “user experience” = {Q1,Q2,Q3}, and “family medicine” = {Q7, Q8}. The same color-coding of the LS-Scores as in Fig. 2 is employed (5 = green, 4 = light green, 3 = gray, 2 = orange, 1 = red, "not applicable" = white)

In order to dissect the user opinion, we further clustered all questions (Table 1) into five categories: (1) “research” = {Q5,Q9}, (2) “perioperative use” = {Q10}, (3) “home monitoring” = {Q4,Q6,Q11}, (4) “user experience” = {Q1,Q2, Q3 }, and (5) “family medicine” = {Q7,Q8}. Our results are summarized by the stacked barplot in Fig. 4.

The comparison of different LS-Score distributions demonstrates (Fig. 4), that the participants of our study showed the most positive scoring in the category "research" (LS-Score 4.5, on average). However, the category "user experience" also exhibited a very high score (LS-Score 4.4, on average) with subsequently high agreement rate of 84.8%. These scores further did not show significant differences when further subdividing the comfort in daytime (Q1) and nighttime use (Q2) of the fitness trackers (*p* = *0.51*). Moreover, also in the category "family medicine", we observed no significant difference (*p* = *0.48*) between the patients' opinions regarding real-time data access by family doctors (Q8) and data access only in the presence of the patient (Q7). Comparing “perioperative use” with “home monitoring” we observed a comparable rate of positive answers for both categories (77.42%; 76.77%). A more detailed analysis of the questions on the disadvantages (Q6) and advantages (Q11) of “home monitoring”-fear of permanent monitoring versus an increased sense of safety—shows that Q11 yield besides the higher rate of agreement (81.8 vs. 72.7%), also the higher rate of disagreement (15.2 vs. 12.1%), reflected by LS-Scores of 4.09 and 4.15. These findings can be explained by the higher rate of abstentions in Q6 compared to Q11.

## Discussion

The primary objectives of our study were to assess patients' perceptions of using fitness trackers in healthcare and to evaluate their acceptance. Secondly, we analyzed compliance and the incidence of device associated adverse events. In contrast to most of the previous trials, our participants were equipped with a fitness tracker during their entire hospital stay, to ensure valid results of the survey based on their own user-experience. Our findings suggest that integrating wearable technology into postoperative care is well-tolerated by patients. The overall high number of positive responses (82.1%, Fig. 2) by participants is one of the key factors to paves the way for employing wearable technologies in healthcare.

The continuous, automated, and objective nature of data collection through wearables provides valuable insights with minimal efforts by patients and healthcare professionals, underlining the significance of these devices in medical research as well as patient care [14]. Compared to manual recorded health data, the data quantity and quality can be significantly increased by the use of wearables devices [15]. It has been demonstrated that the continuous monitoring by wearables may help to identify patients who are at greater risk of a prolonged hospitalization or of readmission in acute cardiac patients [10]. Furthermore, first pilot trials already have shown promising results, with technological advancements now enabling the detection of cardiac arrest by wearables [16]. Thereby, wrist-worn devices are the preferred wearable compared to other sensors [17, 18].

The results of our study highlight the high level of patient interest, as reflected by a substantial consent rate of 89.6%. Our observations are in agreement with a similar trial reporting a 86% consent rate [19]. Furthermore, another trial reports a high compliance to wearable technology in postoperative patients [20], reflecting also a common interest among hospitalized patients to participate in monitoring interventions. Moreover, an in-depth analysis of our cohort demonstrates a premature termination in merely 6.1% of the patients, which is substantially lower than the generally anticipated drop out rate of 10% in clinical trials [21]. However, ensuring device safety is paramount: in contrast to another similar study involving 59 subjects with no reported adverse events [4], our study recorded one device associated adverse event (CTCAE Grade I). Therefore, vigilance of health professionals in employing wearable devices remains essential to mitigate such risks, especially when considering that patients with known allergies to any components of the employed devices were already excluded in our study.

Focusing on potential confounders, there was no age-specific significant impact on the answers. Additionally, no confounding effects were detected when assessing the tracker manufacturer, the duration of hospitalization and demographic attributes of the participants. We observed exclusively one significant sex-specific effect, i.e. the wearing comfort during daytime (Q1). As the sizes of the devices employed in our study ranged from 38 to 45 mm, and their weights from 38.8 to 83 g, our results indicate that these differences are not predominant factors affecting user experience.

Patient comfort and user experience are key factors, besides measurement accuracy and regulatory aspects, in implementing mobile sensors into healthcare [17]. Overall, 84.8% of our patients reported a positive user-experience. Furthermore, our findings are in line with a study showing a high patient compliance in wearing mobile sensors both pre- and postoperatively [22]. We observed no general difference in user experiences between a daytime and nighttime use of the attached devices (Q1, Q2), in contrast to the report of a complementary trial investigating the user experience of wrist-worn devices in epilepsy patients [23]. However, our results on the user experience (LS-Score > 4.42) are supporting the results of a survey investigating the user experience with the Fitbit smartwatch (LS-Score 4.36) [20]. Nonetheless, the latter study by Balu et al. reports an average LS-Score of 3.5 when querying patients about their willingness to use wrist-worn trackers, whereas our results suggest that 72.7% of patients supported the use of wearables in any future hospitalization (LS-Score 4.1 in Q10).

In our categorical analysis of the questionnaire, we observed the highest agreement rate (97.0%) for providing wearable data for research (Q9). Privacy concerns remain a crucial aspect of wearable technology integration. Our survey showed that 87.9% of the predominant geriatric patients had little to no data privacy concerns regarding the use of smart sensor technology (Q5), which is in line with another trial investigating data privacy using wearable data in geriatric patients with 81% agreement [24]. Therefore, balancing the security benefits of continuous monitoring with the potential discomfort associated with constant surveillance remains a key consideration for regulatory bodies and manufacturers.

Shifting the focus from in-hospital use to ambulatory care, we further investigated the opinions from participations for using wrist-worn devices at home and for family medicine. Particularly considering elderly patients, wearable data might improve monitoring and treatment of chronic diseases, like chronic obstructive pulmonary disease [25] or atrial fibrillation [26]. Shifting the focus towards ambulatory operations, it has been shown that wearable data can improve prehabilitation of elderly patients [27], and also that it can be efficient in the postoperative rehabilitation in orthopedic patients [28]. Following these considerations, 72.7% of our cohort had little to no concerns about a continuous monitoring of their health metrics (Q6), and 81.8% of our participants even stated that an automated alert system for health emergencies at home would enhance their overall feeling of safety (Q11). Comparing responses to Q6 with those to Q11 revealed no statistically significant differences in the LS-Scores (*p* = *0.89*). However, we observed the highest proportion of negative answers (21.2%) for the question investigating the willingness of using wearables at home (Q4). To further evaluate the patients' opinions employing wearable technology into family medicine, we distinguished between two scenarios: "the family doctor can access the wearable data only in the presence of the patient" (Q7), and "the family doctor can access the data always and in real-time" (Q8). We observed that, while there is a high general willingness to provide wearable data to the family doctor, patients prefer that the physician can access the data only in their presence over real-time access (84.8% positive answers to Q7 *vs*. 75.8% to Q8). The differential attitudes towards these points may impose a balancing between data privacy and improved patient care.

### Limitations

Our study has several limitations: First, we employed a self-designed, non-validated questionnaire in German and subsequently translated into English, which affects the comparability of our findings to those from other studies. In the future, a standardized questionnaire would be desirable. Second, since data for our survey was collected exclusively from patients who used a wearable device during the entire hospital stay, the sample size of our study is relatively small, which limits the generalizability of our results. Third, our study also exclusively included cognitively unimpaired participants. However, in a study investigating wearable compliance among late-stage agitated dementia patients, the authors observed trends similar to our study [29]. Yet, another study examining the feasibility of remote monitoring technologies across all symptomatic stages of Alzheimer's disease led to similar conclusions [30]. This underscores sufficient compliance, regardless of the patients' cognitive status.

Fourth, another key consideration is the time span of device use, which was on average 7.6 days prior to the survey in our study. Consequently, we cannot draw any conclusions about the effects of long-term use of fitness trackers in healthcare. Although the use of wearables has already been demonstrated to be feasible in long-term use [4], the patient compliance typically declines over a prolonged time of application [31]. For instance, in a postoperative study, the patients' compliance decreased from 95.2 to 81% within a period of 90 days [20]. In agreement, a complementary study involving postoperative urological patients concluded that due to a decline in compliance over 30 days, the Garmin Vivofit was deemed unsuitable for health data collection [32]. Fifth, the responses might be biased toward the positive due to social desirability. Additionally, certain potential influencing factors, such as educational level, could not be analyzed because these data were not available for the investigated cohort.

However, it is important to note that this manuscript evaluates only the attitudes and opinions of patients regarding the use of wearables. This is only one aspect that must be considered before the integration of wearables in healthcare. This includes the development of a robust technical validation strategy for measurement accuracy, the advancement of sensor technology, the generation of valid data on the clinical benefits of such systems, as well as numerous regulatory and legal considerations. Furthermore, the manufacturers of the investigated devices state that while some components and features are approved as medical devices, the devices in their current state are "intended for informational use only" and "not intended to replace traditional methods of diagnosis or treatment."

## Conclusion

Our present study reflected an overall positive feedback of the patient experiences employing fitness trackers postoperatively. The results further provides encouraging preliminary findings to further expand efforts in continuous vital sign monitoring. Future research should therefore focus on further aspects like measurement accuracy, addressing regulatory aspects, developing robust validation strategies, and ultimately providing evidence of clinical benefits to overcome the critical barriers to wider clinical implementation of wearables.

## Supplementary Information

Below is the link to the electronic supplementary material.

Supplementary file1 (PDF 133 kb)

## Data Availability

Data is provided within the manuscript or supplementary information files.
