# Supplementary material for: Postoperative use of fitness trackers for continuous monitoring of vital signs: a survey of hospitalized patients
Source: J Clin Monit Comput. 2025 Mar 6;39(5):1077–86. doi: 10.1007/s10877-025-01273-3 (PMC12474713; doi:10.1007/s10877-025-01273-3)
Supplement: Supplementary file 1 — Supplementary file1 (PDF 133 kb) [file 10877_2025_1273_MOESM1_ESM.pdf]

Supplementary Information

Patienten ID:________________________ Randomisationsgruppe:__________

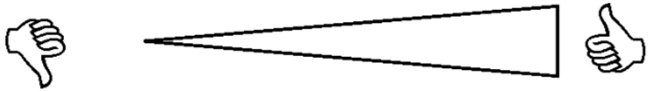

Frage	Enthaltung	Stimme überhaupt nicht zu	Stimme nicht zu	Unentschieden	Stimme zu	Stimme voll zu
**Der Health Tracker hat mich tagsüber nicht gestört.**						
**Der Health Tracker hat mich nachts nicht gestört.**						
**Den allgemeinen Tragekomfort fand ich gut.**						
**Ich würde vergleichbare Geräte auch zu Hause benutzen.**						
**Ich habe keine Bedenken was den Datenschutz angeht.**						
**Die Vorstellung einer dauerhaften Überwachung meiner Gesundheitswerte macht mir keine Angst/Unbehagen.**						
**Ich würde mir wünschen, dass mein Hausarzt die Messwerte in meiner Anwesenheit abrufen kann.**						
**Ich würde mir wünschen, dass mein Hausarzt die Messwerte in Echtzeit abrufen kann.**						
**Ich wäre bereit Messdaten von Health Trackern der Forschung zur Verfügung zu stellen.**						
**Ich würde mir wünschen, auch bei einem zukünftigen Krankenhausaufenthalt Health Tracker zu verwenden.**						
**Ich würde mich mit automatisierten Alarmierungssystemen für gesundheitliche Notfälle zu Hause sicherer fühlen.**						

______________________ ______________________

Datum Unterschrift (Studienpersonal)
